# Examining the Diagnostic Yield of Tumour Testing and Qualifying Germline Concordance for Hereditary Cancer Variants in Patients with High-Grade Serous Carcinoma

**DOI:** 10.3390/genes13081398

**Published:** 2022-08-06

**Authors:** Emily A. Goebel, Jennifer Kerkhof, Oleksandra Dzyubak, C. Meg McLachlin, Jacob McGee, Bekim Sadikovic

**Affiliations:** 1 Department of Pathology and Laboratory Medicine, London Health Sciences Centre, London, ON N6A 5A5, Canada; 2 Department of Pathology and Laboratory Medicine, Schulich School of Medicine and Dentistry, Western University, London, ON N6A 5C1, Canada; 3 Verspeeten Clinical Genome Centre, London Health Sciences Centre, London, ON N6A 5W9, Canada; 4 Department of Obstetrics and Gynecology, London Health Sciences Centre and Schulich School of Medicine and Dentistry, Western University, London, ON N6A 5W9, Canada

**Keywords:** ovarian carcinoma, BRCA, molecular testing, solid tumour

## Abstract

Despite advances in treatment, prognosis for most patients with high-grade serous carcinoma (HGSC) remains poor. Genomic alterations in the homologous recombination (HR) pathway are used for cancer risk assessment and render tumours sensitive to platinum-based chemotherapy and poly (ADP-ribose) polymerase inhibitors (PARPi), which can be associated with more favourable outcomes. In addition to patients with tumours containing *BRCA1* or *BRCA2* pathologic variants, there is emerging evidence that patients with tumours harbouring pathologic variants in other HR genes may also benefit from PARPi therapy. The objective of this study is to assess the feasibility of primary-tumour testing by examining the concordance of variant detection between germline and tumour-variant status using a custom hereditary cancer gene panel (HCP). From April 2019 to November 2020, HCP variant testing was performed on 146 HGSC formalin-fixed, paraffin-embedded tissue samples using next-generation sequencing. Of those, 78 patients also underwent HCP germline testing using blood samples. A pathogenic variant was detected in 41.1% (60/146) of tumours tested, with 68.3% (41/60) having either a *BRCA1* or *BRCA2* variant (*n* = 36), or *BRCA1/2* plus a second variant (*n* = 5), and 31.2% (19/60) carrying a pathogenic variant in another HCP gene. The overall variant rate among the paired germline and tumour samples was 43.6% (34/78), with the remaining 56% (44/78) having no pathogenic variant detected in the germline or tumour. The overall *BRCA1/2* variant rate for paired samples was 33.3% (26/78), with germline variants detected in 11.5% (9/78). A non-*BRCA1/2* germline variant in another HCP gene was detected in 9.0% (7/78). All germline variants were detected in the tumour, demonstrating 100% concordance. These data provide evidence supporting the feasibility of primary-tumour testing for detecting germline and somatic variants in HCP genes in patients with HGSC, which can be used to guide clinical decision-making, and may provide opportunity for improving patient triage and clinical genetic referral practices.

## 1. Introduction

*BRCA1/2* germline testing in patients with high-grade epithelial ovarian carcinoma (EOC) is now considered the standard of care [1]. Germline genetic testing in patients with EOC enables hereditary cancer detection that triggers specific cancer-prevention strategies and the genetic testing of family members [2,3]. In addition, patients with germline and somatic *BRCA*-mutated high-grade EOC are eligible for poly (ADP-ribose) polymerase inhibitor (PARPi) maintenance therapy [4]. Several trials have shown that PARPi maintenance therapy can prolong progression-free survival in ovarian cancer patients [5,6,7,8].

Tumour testing can identify somatic variants, independent of germline status [1,9]. In contrast to germline testing, which identifies inherited variants, tumour testing enables the identification of both germline and somatic variants and therefore expands potential eligibility for therapeutics [10,11]. Specifically, tumour testing for *BRCA1/2* variants can identify more patients who might be eligible for PARPi treatment [3,12,13,14]. Germline *BRCA1*/*2* pathogenic variants occur in 22.6% of high-grade serous carcinomas (HGSCs), while somatic *BRCA1/2* pathogenic variants have been shown in an additional 6–7% of HGSCs [15]. Thus, all patients with a *BRCA1/2* pathogenic variant (germline or somatic) can benefit from PARPi therapy after the completion of front-line chemotherapy.

In 2016, the use of PARPi for recurrent high-grade epithelial carcinoma of the ovary, fallopian tube, and peritoneum was approved for patients with *BRCA1/2* germline or somatic pathogenic variants in Canada [16]. This approval led to the increased utilisation of genetic testing, and a proposal for reflexive tumour testing to provide actionable information for treatment purposes was made [17]. Moreover, because of the increasing shortage of genetic counselling capacity, a more focused germline testing approach is needed [11,18]. Reflex tumour testing could improve the efficiency of this process by focusing genetic counselling referrals on patients with somatic variants in hereditary cancer genes. A recent study comparing *BRCA1/2* variant status between germline and somatic testing results showed 100 percent concordance, providing validation for the use of tumour testing to the determine potential utility of treatment, as well as hereditary cancer risk [19].

While germline and somatic *BRCA1*/*2* variants are currently considered the most clinically relevant, patients with variants in other HR pathway genes (either in germline or tumour) may also benefit from PARPi therapy [13]. It is therefore necessary to establish a robust method for detecting these variants. While Ong et al. analysed several other variants, including *ATM*, *PALB2*, *TP53*, and *APC*, in tumours from patients with HGSC [20], no studies have investigated the concordance rate of a more comprehensive panel of hereditary cancer genes between tumour and germline testing.

The objective of this study is to examine the concordance of hereditary cancer gene variants between germline and tumour testing in patients with HGSC using an institutional hereditary cancer gene panel (HCP) of 37 genes. We aim to demonstrate the feasibility of primary-tumour testing and assess the extent to which tumour testing can reliably capture germline pathogenic cancer gene variants.

## 2. Materials and Methods

### 2.1. Study Population

The study population included a retrospective cohort of all patients with HGSC at our institution from April 2019 to November 2020. Genetic tumour testing was previously performed on patient samples; this consisted of a clinically validated 37-gene Hereditary Cancer Panel (HCP), which enabled the simultaneous detection of both sequence and copy-number variations (CNV) of the target genes using next-generation sequencing (NGS). Germline testing was also previously reported and blinded prior to the assessment of tumour specimens. Tumour test results were compared with matched germline testing results, for those available. Access to funded genetic testing in Ontario is limited to patients meeting the criteria defined by government regulations, and therefore, only a subset of cases had previous germline testing results available.

### 2.2. Sample Preparation and Testing

#### 2.2.1. Hereditary Cancer Panel

The previously clinically validated 37-gene HCP examined all coding exons and 20 bp of flanking intronic sequences for the 37 genes (File S1), and was designed to achieve a > 500X mean read depth coverage and a minimum 100X coverage at a single-nucleotide resolution. HCP is used in the clinical setting for more than one referral indication and filtered for the genes requested as an effective way to test for multiple indications. For clinical referrals of EOC, *BRCA1/2* are assessed only. *TP53* is included in the HCP, but was not assessed for the purposes of this study. The sensitivity detection of this custom NGS pipeline has been validated previously for minor allele detection levels at 2–5%, along with sub-exon-level CNV detection [21].

#### 2.2.2. DNA Isolation

Three 20 μm sections from formalin-fixed paraffin-embedded (FFPE) tissue samples with adequate tumour cellularity were obtained for DNA extraction using a sterile protocol. Genomic DNA was isolated using the Invitrogen RecoverAll total nucleic acid isolation kit (Thermo Fisher Scientific, Waltham, MA, USA), according to the manufacturer’s protocol.

Genomic DNA from each peripheral blood sample was isolated by standard protocols using the MagNA Pure system (Roche Diagnostics, Laval, QC, Canada).

#### 2.2.3. Next-Generation DNA Sequencing (NGS)

NGS libraries were prepared as described previously [22,23]. Briefly, 100 ng of fragmented genomic DNA was ligated with a specific barcode and pooled with 23 other sample libraries for a 24-plex run that was captured using the SeqCap EZ Choice Library system according to the manufacturer’s protocol (Roche NimbleGen, Inc., Madison, WI, USA). Captured libraries were diluted to 8 pM or 1.3 pM for sequencing with the MiSeq v2 or NextSeq v2.5 mid output kits, respectively (Illumina, San Diego, CA, USA). Sequencing reads were generated as 2 × 150 bp paired-end reads with post-sequencing file conversion to FASTQ for alignment with NextGene software version 2.4.2.3 (SoftGenetics, LLC, State College, PA, USA) using standard alignment settings. Variants were filtered at an allelic fraction of > 10% to minimise the impact of sequence artifacts and mutational burden and were classified by a clinical molecular geneticist based on the College of American Pathologists (CAP) and the American College of Medical Genetics and Genomics (ACMG) standards and guidelines for pathogenicity [24,25]. For this study, all assessed Tier I/II variants (variants of strong and potential clinical significance (therapeutic, prognostic and diagnostic)) [24] and ACMG 1/2 variants (pathogenic or likely pathogenic variants) [25] were reported.

#### 2.2.4. Detection of Copy-Number Variants by NGS

Base coverage distribution reports were created using NextGene software (SoftGenetics, LLC) and processed through a normalisation algorithm described previously [22,23]. CNV assessment was performed through quantile normalisation for all 37 genes on the HCP to eliminate ambiguous findings. Detected CNVs were then filtered for the genes of interest prior to assessment. For FFPE samples, the limit of detection of whole gene deletions for *BRCA1* or *BRCA2* was 30%. Sub-gene-level events were identified by a minimum of 50% deviation from the nomalised values of the remainder of the gene.

### 2.3. Data Analysis

Results are reported using descriptive statistics. For samples in which tumour and matched germline testing was available, variant identification was compared to determine concordance. In order to assess if tumour analysis could further direct germline assessment, type of variants by origin, tumour variant allelic fraction (VAF) based on origin, and sequence variant origin based on gene distribution were examined.

## 3. Results

### 3.1. Tumour Testing Analysis

**Figure 1:**
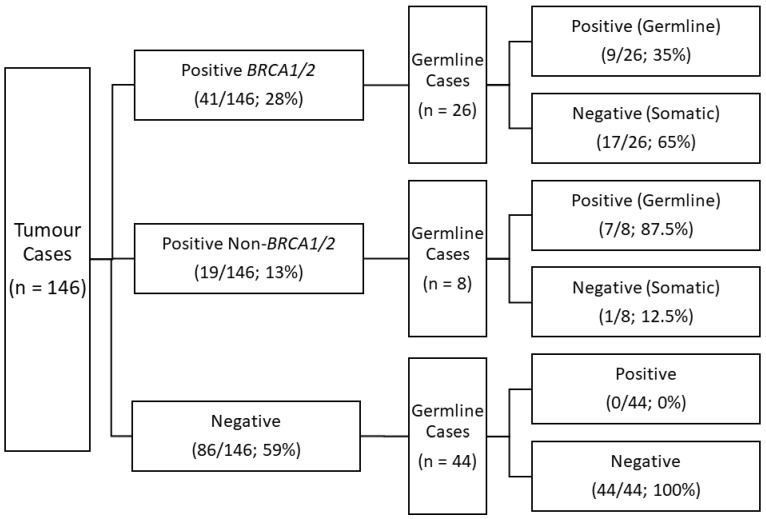
Summary of HGSC tumour and matched germline results. Tumour samples were divided into three groups: 1. Tumour cases testing positive for a *BRCA1* or *BRCA2* Tier I/II variant; 2. Tumour cases testing positive for a Tier I/II variant in an HCP gene excluding *BRCA1*, *BRCA2*, or *TP53*; and 3. Tumour cases with no detected Tier I/II variant. Cases that carried a *BRCA1* or *BRCA2* variant, as well as a second variant in another gene, were classified as *BRCA1/2*-positive. Of the cases with tumour results, matched germline analysis was assessed in a subset of cases. Tumour variants that were also present in germline analysis were deemed inherited while all tumour variants absent from germline analysis were considered somatic. Matched negative tumour cases showed 100% concordance.

A total of 150 tumour samples from patients with HGSC were received for somatic tumour testing during the 19-month study period; however, 4 were removed from analysis due to an insufficient amount of DNA extracted (*n* = 1) and duplicate specimens received (*n* = 3). Of the 146 FFPE HGSC tumour specimens tested, 41 (28%) carried a Tier I/II variant in either *BRCA1* or *BRCA2* (*n* = 36), or *BRCA1/2* plus a second variant (*n* = 5). An additional 19 (13%) specimens carried one or multiple Tier I/II variants in the remaining HCP genes: *APC* (*n* = 1), *ATM* (*n* = 1), *BARD1* (*n* = 1), *BRIP1* (*n* = 1), *CDKN2A* (*n* = 2), *MSH3* (*n* = 3), *MUTYH* (*n* = 4), *NBN* (*n* = 1), *PALB2* (*n* = 1), *PMS2* (*n* = 1), *POLE* (*n* = 2), *PTEN* (*n* = 3), *RAD51C* (*n* = 1), *RAD51D* (*n* = 2), and *SDHB* (*n* = 1). A complete list of variants identified are available in Table S1. The remaining 86 (59%) tumour specimens showed no evidence of Tier I/II variants in any of the 36 HCP genes tested (Figure 1).

### 3.2. Germline Testing Analysis

A total of 78 tumour specimens had matching germline assessments completed (Figure 1). Cases in which tumour variants were identified in germline DNA (*n* = 16; 20.5%) were labelled as germline-positive while ones without germline variants (*n* = 18; 23.1%) represented somatic events. 

In the *BRCA1/2* tumour-positive cohort, 26 cases had matching germline assessments, with 9 (35%) being a germline event and 17 (65%) representing a somatic event. One of the somatic cases (Case 031) carried two *BRCA1* variants and both were deemed somatic in origin (Table S1). Another somatic case (Case 060) carried a *BRCA2* somatic variant, but also a germline *MUTYH* variant (Table S1). The non-*BRCA1/2* tumour cohort showed that 7/8 (87.5%) were of germline variant origin and 1/8 (12.5%) was a somatic event (Figure 1). Two of the germline cases (Case 014, 016) identified one germline and one somatic variant, while an additional case (Case 017) showed two germline variants (Table S1). Overall, germline assessment identified 18 ACMG 1/2 variants, with 100% concordance in tumour analysis. Germline assessment of the negative tumour cohort was performed on 44 cases, and all were negative by germline analysis, indicating that the tumour assessment was 100% sensitive for the detection of germline variants (Figure 1).

### 3.3. Variant Origin Analysis

**Figure 2:**
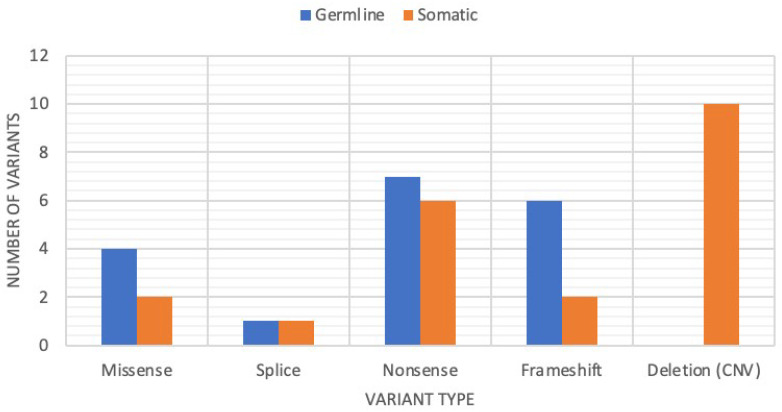
Distribution of variant type based on variant origin. Large full-gene copy-number variation (CNV) deletion events were only observed as somatic in origin while all other variant types were observed across both types of inheritance.

Although there were a greater number of somatic variants (*n* = 21) than germline variants (*n* = 18), all types of sequence variants were represented across both variant origins. However, all large, whole-gene deletion events (*n* = 10) were observed as somatic in origin (Figure 2). This copy-number pipeline is designed to detect sequence CNVs; however, the span of the copy-number alteration beyond the NGS target gene locus and the type of the chromosomal structural abnormality cannot be determined.

**Figure 3:**
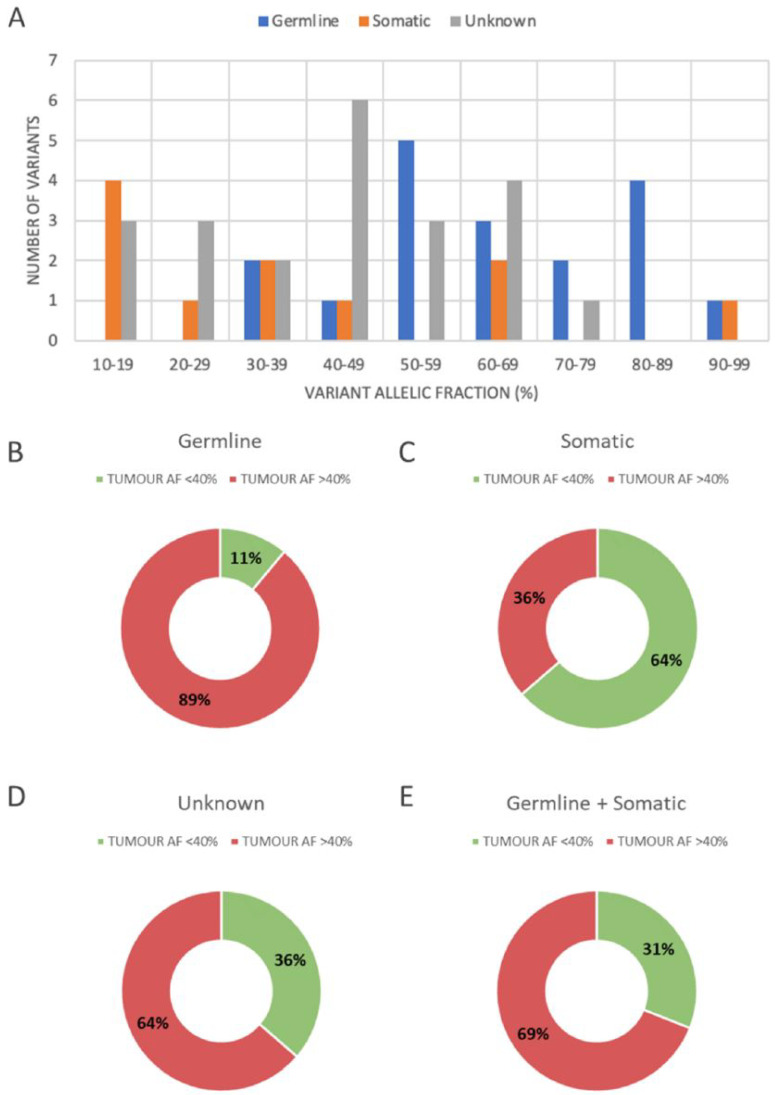
Distribution of sequence variant origin based on tumour variant allelic fraction (VAF). The number of variants of somatic, germline and unknown origin with tumour VAFs are shown in (**A**). The proportion of variants with a VAF greater than and less than 40% for germline origin (**B**) demonstrates that germline variants are more likely to have a high VAF, while the opposite is true for somatic variants (**C**). The distribution of variants that were present in tumour analysis, but did not have matching germline analysis (**D**) show a distribution very similar to the combined germline plus somatic origin (**E**).

Germline sequence variants demonstrated a tumour VAF ranging from 33.3% to 96.3%, with 16/18 (89%) showing a VAF greater than 40% (Figure 3A,B). Somatic sequence variants demonstrated a tumour VAF ranging from 11.4% to 90.5%, with 8/11 (64%) showing a VAF of less than 40% (Figure 3A,C). The distribution of variants of unknown origin (tumour assessment only) paralleled that of the combined germline and somatic variants (Figure 3D,E). All germline variants had a tumour VAF greater than 30%.

**Figure 4:**
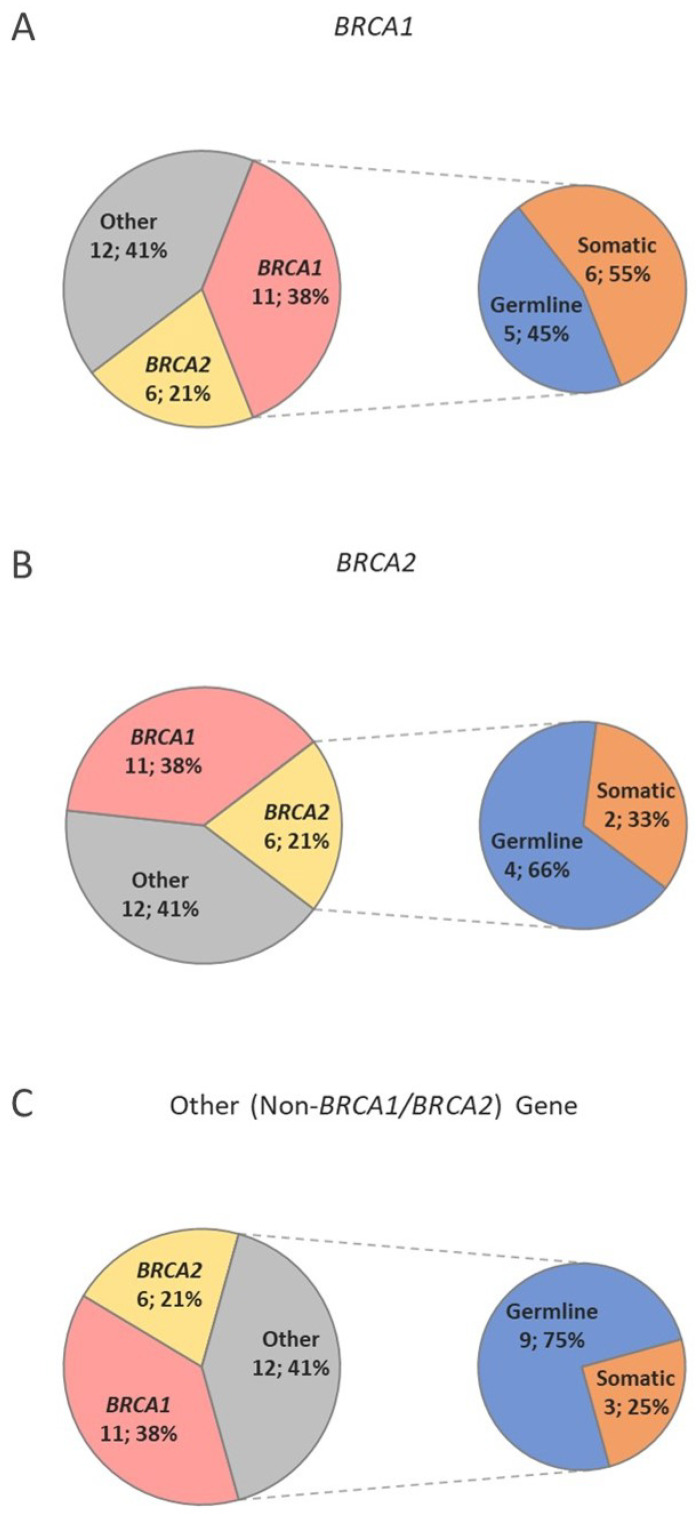
Distribution of all sequence variants between *BRCA1*, *BRCA2* and another HCP gene (*APC*, *BRIP1*, *CDKN2A*, *MSH3*, *MUTYH*, *PALB2*, *PTEN*, *RAD51C,* and *RAD51D).* Distribution of variant origins are demonstrated for *BRCA1* (**A**), *BRCA2* (**B**), and other HCP genes (**C**). The other category demonstrates a high proportion of germline variants that would not be detected with isolated *BRCA1* and *BRCA2* tumour analysis.

Finally, we assessed sequence variant origin based on gene distribution. *BRCA1* sequence variants accounted for 11/29 (38%) variants and 5/11 (45%) were of germline origin (Figure 4A). *BRCA2* sequence variants accounted for 6/29 (21%), and 4/6 (67%) were of germline origin (Figure 4B). The other (non-*BRCA1*/*2*) HCP genes, which accounted for 12/29 (41%) and 9/12 (75%), were of germline origin (Figure 4C).

## 4. Discussion

Our results indicate that EOC tumour testing can detect both *BRCA1/2* Tier I/II and ACMG 1/2 variants. In this patient cohort, representing a retrospective assessment of sequential patient samples that were clinically tested in a tertiary hospital setting using the standardized clinical assessment protocols, no variant was detected in the germline that was not detected by tumour testing. On the contrary, tumour testing identified somatic variants in 23.1% of patients without germline variants. Of these, *BRCA1/2* variants were the most common (17/78, 21.8%), rendering patients eligible for PARPi therapy.

In terms of somatic tumour testing, our results mirror those of Fumagalli et al., who found that 5/23 (21.7%) pathogenic/likely pathogenic variants were identified through tumour testing only and would not have been detected using germline testing alone [10].

In addition to expanding patient eligibility for access to targeted therapy, tumour testing, which can be performed reflexively as part of the routine pathology assessment at the time of diagnosis, has implications for hereditary cancer syndrome detection. In Canada, there are significant wait times for genetic counselling and reported referral rates can be extremely low (6.6%) [26]. However, since the implementation of reflex tumour testing for *BRCA1/2* variants, one institution has seen improvements in the rate of genetic referral (12.88% versus 7.10%) and time to genetic counselling appointment (59 days versus 33 days) [27]. Similarly, our study shows that tumour testing can be used to triage patients for genetic counselling by prioritizing those with a positive tumour result, since all germline-positive patients were also positive by tumour testing. Previously, our institution has shown that high genetic counselling referral rates (>99%) can be achieved through a direct referral pathway [28]; however, this puts significant burden on clinical genetics resources and triaging patients based on tumour testing has the potential to ensure only those with increased genetic risk are referred.

Our study shows that tumour testing can be performed to detect pathogenic variants in other HR pathway genes. Importantly, several studies have reported the efficacy of PARPi in patients with non-mutated BRCA high-grade EOC [29,30,31,32,33,34] and data from randomised controlled trials indicate that when compared with placebo, PARPi therapy improves progression-free survival in patients with HRD-positive tumours, and the degree of PFS benefit was greater in this group compared to patients with *BRCA* wild-type and HRD-negative tumours [7,35,36]. As such, in addition to *BRCA1/2*, other genes in the HR repair pathway, which may provide information for more complete ovarian cancer management, should also be analysed in tumour samples. Specifically, non-*BRCA1/2* variants were identified in an additional 13% of tumour samples in our study (*APC*, *BRIP1*, *CDKN2A*, *MSH3*, *MUTYH*, *PALB2*, *PTEN*, *RAD51C,* and *RAD51D)*.

At this time, in Ontario, only *BRCA1/2* testing is mandated for the determination of PARPi therapy eligibility, and as such, tumour testing cannot supplant germline testing. As reported by others, and reiterated in our study, tumour testing for *BRCA1/2* variants is a robust way to triage patients with *BRCA1/2* variants, not only for PARPi therapy, but possibly also for genetic counselling [10,37]. Recognizing that non-*BRCA1/2* variants still have clinical relevance, until tumour testing is expanded, germline testing cannot be replaced. The American Society of Clinical Oncology (ASCO) guidelines recommend multigene panel germline testing for patients with ovarian carcinoma; this includes, at least, B*RCA1*, *BRCA2*, *RAD51C*, *RAD51D*, *BRIP1*, *MLH1*, *MSH2*, *MSH6*, *PMS2*, and *PALB2,* as these have been associated with the risk of inherited ovarian cancer [1]. This could have implications for further cancer screening in patients and risk-reduction strategies, such as salpingo-oophorectomy, in at-risk family members [38]. In our study, germline mutations in *RAD51C*, *RAD51D*, *BRIP*, *MSH3*, *APC*, *MUTHY*, and *PALB2* were identified. In addition to the association between inherited ovarian cancer and *RAD51*, *BRIP*, and *PALB2* pathogenic variants, *MUTHY* pathogenic variants confer an increased risk of ovarian cancer and may demonstrate resistance to platinum-based agents, much like tumours with mismatch repair deficiency [39]. Our study provides further support for the expansion of tumour testing to include other HCP genes, as a higher proportion of non-*BRCA1/2* variants detected in tumours were of germline origin. In addition, expanding tumour testing may offer further information for tailoring targeted therapies.

Examining *BRCA1/2* VAF to gain insights on variant origin has been previously examined [19]. While a study at a single institution found that the VAF in all germline *BRCA1/2* pathogenic variants was over 40% (44–94%), there was a wider range in VAF for variants of uncertain significance (5–90%) [19]. In our study, using a VAF cut-off of 40% would have identified nearly, but not all, germline variants (89%); this urges further caution that relying on VAF cut-offs may miss important groups. All germline variants in our study had a tumour VAF greater than 30%, indicating a possible cut-off value for referral to follow-up germline testing. As there is pressure to streamline the flow of genetic testing in Ontario, setting this value would minimise the number of referrals to clinical genetics, and it is likely to capture all potential germline cases. However, since we demonstrated 100% sensitivity for the detection of germline variants by tumour testing, we could still decrease the number of HGSC genetic counselling referrals by nearly 60% if we referred all cases with a positive tumour genetic profile, regardless of VAF. Although there is support for suggesting tumour testing as the initial screen for the detection of potential germline variants, it is still important to consider personal and family history in those with a negative genetic tumour profile.

There are some general limitations to this study. First, the HCP utilised was a 37-gene panel; further expansion of the panel may detect other relevant variants. Moreover, although included in the HCP, *TP53* analysis was not reported on tumour samples as the pathogenesis of HGSC is driven by p53 dysfunction and *TP53* pathologic variants are present in almost all HGSCs [40]. Therefore, any potential *TP53* germline variants were not reported. As one of the goals of this study is to provide support for tumour testing as a potential way to triage patients for genetic counselling and follow-up germline testing, including *TP53* pathologic variants would flag high numbers of patients for further testing and would not be practical in our healthcare system. Second, this was a retrospective study of a 146-patient cohort; prospective studies with a larger patient cohort will provide further insight into the utility of tumour genetic testing for the purposes of therapy eligibility and genetic testing referrals. Studying FFPE tissue also comes with limitations due to the quality of the DNA specimens obtained, which can impact the detection of more complex variants, such as CNVs. The detection of all CNVs in this study were of somatic origin and mostly full-gene events. This could indicate the involvement of larger chromosomal rearrangements that were not assessed in this study. Without the assessment of any germline cases that carry a large CNV, additional studies are needed to determine the feasibility of primary detection in tumour specimens before the adaptation of this workflow. Alternatively, supplementary techniques, such as MLPA, could be performed in parallel to rule out any CNVs. Finally, the limit of detection for somatic variants in this study was 10%, but it has not been evaluated if those with a lower tumour heterogeneity could also benefit from targeted PARPi therapy.

The failure rate of tumour testing was low at our institution (0.67%), and reliable results were obtained from tumour samples following neoadjuvant chemotherapy (33.3% of cases). Proof of an efficient and robust tumour testing pathway has several advantages in clinical practice. First, by examining the molecular characteristics of a tumour at the time of diagnosis, timely and appropriate therapeutic decision-making can be made. In the context of HGSC, somatic *BRCA1/2* variants allow for the identification of more patients who would be suitable for PARPi therapy. Additionally, tumour testing has the potential to enhance the efficiency of genetic testing. Because both germline and somatic cancer variants can be detected in tumours, tumour testing could act as an initial screen for eligibility for germline testing and could potentially replace initial germline testing. Lastly, for deceased patients with HGSC who did not undergo *BRCA1/2* genetic testing, testing of archived tumour tissue to detect possible germline HCP variants could provide essential information to family members.

## 5. Conclusions

This study provides support for reflex tumour testing with a comprehensive NGS panel that includes *BRCA1/2* and other HR genes in order to determine treatment eligibility and aid in triaging patients for germline testing and genetic counselling referrals. Further studies are required to examine the concordance of variant detection in tumour tissue, normal tissue, and germline to help further determine the validity of tissue testing directly as an initial screen for germline variant detection.

## Data Availability

In accordance with the journal’s guidelines, data are available upon reasonable request.

## Supplementary Materials

The following supporting information can be downloaded at: https://www.mdpi.com/article/10.3390/genes13081398/s1, File S1: 37-gene Hereditary Cancer Panel; Table S1: Variants identified in HGSC tumour specimens.
